# Health system strengthening is needed to respond to the burden of pain in low- and middle-income countries and to support healthy ageing

**DOI:** 10.7189/jogh.09.020317

**Published:** 2019-12

**Authors:** Saurab Sharma, Fiona M Blyth, Shiva Raj Mishra, Andrew M Briggs

**Affiliations:** 1 Kathmandu University School of Medical Sciences, Dhulikhel, Nepal; 2 Dunedin School of Medicine, University of Otago, Dunedin, New Zealand; 3 Concord Clinical School Faculty of Medicine and Health, University of Sydney, Sydney, Australia; 4 Nepal Development Society, Chitwan, Nepal; 5 School of Physiotherapy and Exercise Science, Curtin University, Perth, Australia

## THE IMPACT OF PAIN IN LOW- AND MIDDLE-INCOME COUNTRIES

**Figure 1:**
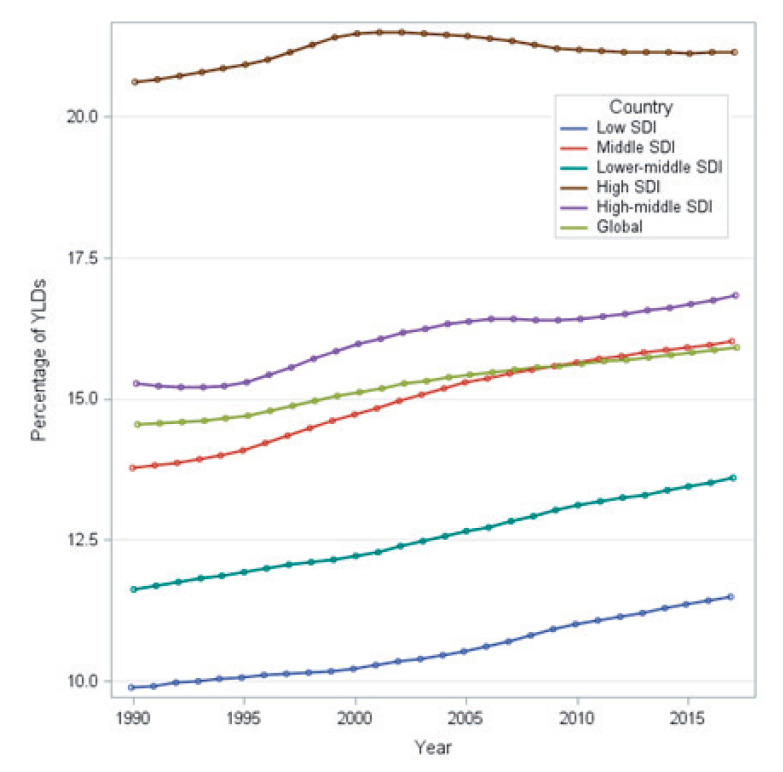
Burden of disease for musculoskeletal in people aged 50-69 years [2]. YDL – years lived with disability, SDI – sociodemographic index.

Although pain is experienced across the life course, the prevalence, particularly of musculoskeletal pain, increases with age [1]. Musculoskeletal pain disproportionately affects people in low- and middle-income countries (LMICs) and the associated disease burden continues to rise [2]. About 25% of the general population in LMICs experience chronic musculoskeletal pain, and this estimate increases by two to four times among working populations [1]. Musculoskeletal conditions represented the leading cause of disability in people aged 50-69 years among low-middle sociodemographic index (SDI) countries in the 2017 Global Burden of Disease (GBD) study and the second leading cause in people aged 70 years and over (**Figure 1**) [2]. Pain management, particularly for musculoskeletal conditions, is necessary for improving quality of life and enabling functional ability, work and participation to avoid poverty. In the context of rapid global ageing, particularly in LMICs [3], system strengthening approaches for pain management are urgently needed to respond to this global health burden.

## DRIVERS OF AN INCREASING PAIN BURDEN IN LMICS

A myriad of factors contribute to the increasing burden of pain in LMICs. First, most LMICs are now facing sociodemographic and health challenges previously considered problems of high-income countries (HICs). Disproportionately rapid ageing populations in LMICs are more prone to frailty, sarcopenia and falls-related injuries such as low-trauma fractures due to bone fragility [3]. LMICs are also experiencing an increasing burden of NCDs and their risk factors (physical inactivity, poor nutrition, pollution and substance abuse) and injury-related pain particularly due to road traffic accidents and violence, all on a background of an existing substantial burden of communicable diseases [2]. More complex health presentations associated with ageing, characterised by multimorbidity of NCDs, are increasingly common in LMICs [4] and musculoskeletal pain conditions are a very common component of multimorbid health profiles. Similarly, neuropathic pain is common in neurological conditions such as stroke, spinal cord injuries, and multiple sclerosis; communicable diseases such as AIDS/HIV, leprosy, and herpes zoster; and genetic disorders such as sickle cell anaemia [5]. LMICs experience additional burden from higher rates of injuries related to trauma, violence and natural disasters compared to HICs [2], which further predispose people to long-term pain-related problems across the life course.

Second, work in LMICs is disproportionately physically demanding, especially in rural areas and lower socioeconomic groups. Work-related pain is accentuated in LMICs by longer hours of work in fields or factories, manually ploughing fields, and carrying heavy loads for extended periods in difficult terrains.

Third, access to pain management services in LMICs is limited and available health services are ill-equipped to manage case complexity. Limited access to health care and health information, limited skills among health workers for treating pain, limited long-term care systems for older adults, and inadequate support for optimising self-care are notable challenges in LMICs [3], which point to the need for a system strengthening approach to pain care in these settings.

## PAIN MANAGEMENT

Untreated pain not only threatens healthy ageing and contravenes the 2010 Declaration of Montréal, which advocated that access to pain relief is a fundamental human right; but also represents a broader threat to achieving targets of the Sustainable Development Goals (SDGs) including poverty and economic growth. Human suffering from unrelieved pain is a major global health problem, particularly in LMICs, where many terminally ill patients and patients with acute pain do not receive essential analgesic medications [6]. For example, 71% of the world’s population has an extremely low consumption of opioids for acute pain relief [6]. In contrast, HICs consume 79% of global morphine stock, including when often not indicated, for example, in chronic non-cancer pain (CNCP) [6]. Management for CNCP, typically of musculoskeletal origin, varies to the management approach for acute pain. First-line treatment consists of education including reassurance, staying employed in productive work, supporting graded activity, psychological therapies, among others [7]. For many people with chronic and disabling pain, multidisciplinary care is needed, but rarely accessible in LMICs.

**Figure:**
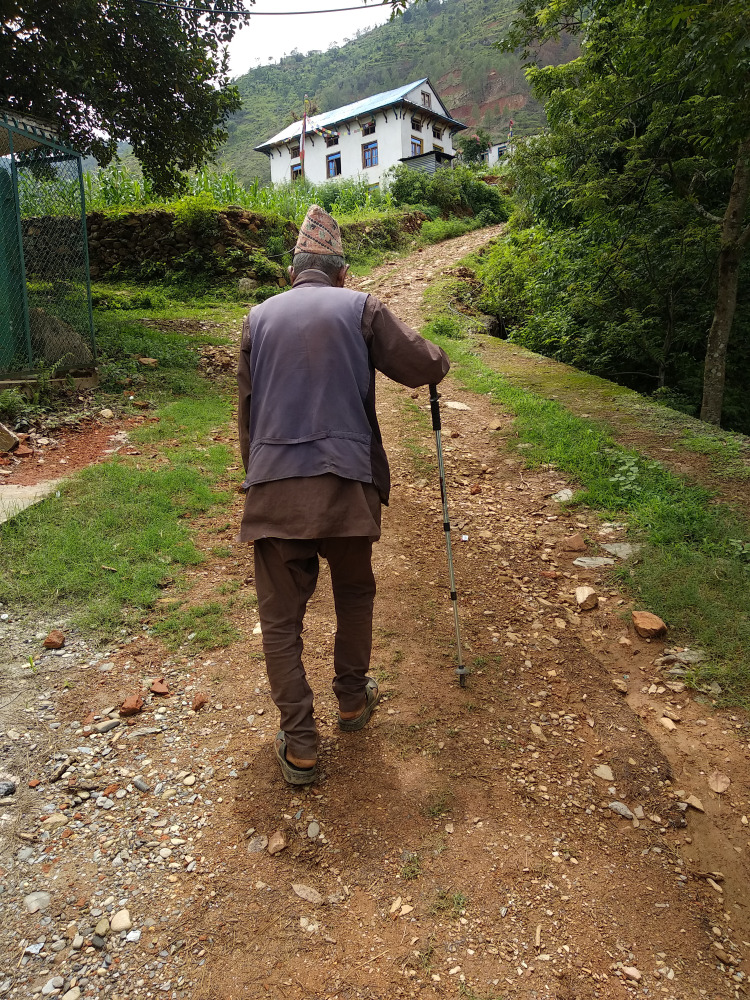
Photo: From the collection of Manisha Shrestha, BPT in Bolde, Nepal (used with permission).

## RESPONDING TO THE BURDEN OF PAIN IN LMICS

Considering the magnitude and burden of pain in LMICs, strong and comprehensive system-level responses are required to positively reform health and social care services and systems, integrating with other global system-strengthening initiatives that target population health needs in ageing, long-term care, rehabilitation, palliative care, and prevention and control of NCDs. The 2020-2030 Decade of Healthy Ageing and 2030 targets for the SDGs provide an opportune time to prioritise pain care in LMICs.

### Public health and service delivery

Population-level public health interventions such as mass media campaigns can be effective and resource-efficient interventions for a large-scale public health problem such as low back pain [8]. An evidence-informed pain education programme was recently developed in Nepal using local contexts for preventive, curative, and rehabilitative approaches for pain conditions, which can be used in population health campaigns using various delivery methods [9].

Access to community-based and effective health services for pain management is needed, particularly for older people. The World Health Organization (WHO) Integrated Care for Older People (ICOPE) approach provides a service-level framework to guide the implementation of community-based health services, where musculoskeletal health is considered a key determinant of healthy ageing [10].

### Access to essential analgesic medicines and rehabilitation services

While access to essential analgesic medicines for palliative care, post-surgical pain, acute injury and childbirth is often limited or sometimes unavailable in LMICs [6], paradoxically some individuals with chronic pain conditions in the same LMICs receive opioid analgesics when they are not appropriate or safe – having been associated with poor outcomes including no functional improvement, hyperalgesia and death. Although calls for access to pain relief in LMICs have largely focused on availing opioids for essential pain relief for acute pain, cancer and palliative care, extrapolating this call of making opioids available in LMICs for CNCP is common and unhelpful, highlighting the need for appropriate regulation. Based on the experiences in HICs, particularly in the United States, this may worsen the disability burden associated with CNCP in LMICs.

One viable alternative to avoid overuse and adverse effects of opioids/analgesics in CNCP is improving multidisciplinary rehabilitation services. Rehabilitation services are increasingly important in LMICs because of increasing life expectancies with an increasing number of functional impairments related to musculoskeletal pain conditions. Additionally, the current rehabilitation services in the LMICs, if at all available, are not adequate to meet the rehabilitation needs of the ageing population [3]. To meet this disparity, the WHO Rehabilitation 2030 initiative has called for improvement to global rehabilitation services by 2030 (https://www.who.int/rehabilitation/rehabilitation_health_systems/en/). Provision of appropriate rehabilitation services as a component of universal health coverage (UHC) for older people is essential not only to improve individuals’ functional levels and community participation, but will contribute to the achievement of the SDG targets for health and poverty.

### Health workforce

A workforce (ideally community-based) of adequate volume and competencies is needed to address the burden of pain in LMICs. Here, workforce training and broadening of work cadres, such as community-based health workers, will be important to address population needs [10]. For LMICs, upskilling community-based health workers and carers in appropriate pain care will be essential, as well as the implementation of human resource systems to support the paid and unpaid workforce and plan for future workforce needs. It is important to co-design these services with health professionals delivering the interventions and patients receiving them so that the services are accepted by both of these end-users.

### Health information systems

Many LMICs countries currently lack population surveillance and workforce distribution data, or systems to generate data regarding the population burden of pain conditions. Optimisation of health information systems that include surveillance of pain, as demonstrated recently in the Solomon Islands [11], will assist with current and future population health planning. Use of existing technologies to support health information sharing, such as mHealth, has great potential for application in health surveillance, system navigation and self-management for consumers.

### Financing

In order to maintain the financial sustainability of health systems, financing models must support value-based pain care within UHC essential packages. This may be achieved through a range of regulatory and legislative levers, such as incentivising integrated, multidisciplinary care for people with chronic pain and minimising out-of-pocket expenses through financial protection for socioeconomically deprived people (eg, India’s ‘Modicare’ scheme); harmonising funding between health and social care services to support holistic, person-centred care; and defunding low-value care options. Without radical reform to health financing, LMICs are unlikely to meet SDG 3 targets.

### Leadership and governance

Development, championship and implementation of health policy that includes pain care are needed in LMICs. Critically, single disease or condition-focused health reform initiatives are unlikely to be effective or sustainable in fragile health systems, highlighting the need for integration of pain care within existing or emerging policies for healthy ageing, prevention/management of NCDs, rehabilitation and palliative care. Consumers, civil society, clinicians and health service managers should be supported to help shape the pain care agenda in LMICs to ensure local needs are considered. Existing models of care and strategies for pain care can then be appropriately adapted and shared with LMICs, as required.

## CONCLUSION

Health system strengthening to improve pain care and surveillance should be priorities in LMICs. If given priority commensurate with the burden of disease, it may have positive impacts on healthy ageing, and also in reducing the burden of other common NCDs.
